# Factors associated with COVID-19 vaccine uptake in a US/Mexico border community: demographics, previous influenza vaccination, and trusted sources of health information

**DOI:** 10.3389/fpubh.2023.1163617

**Published:** 2023-07-27

**Authors:** Angel Lomeli, Arleth A. Escoto, Breanna Reyes, Maria Linda M. Burola, Stephenie Tinoco-Calvillo, Isabel Villegas, Ariel S. Cohen, Louise C. Laurent, Linda Salgin, Nicole A. Stadnick, Borsika Rabin, Marva Seifert

**Affiliations:** ^1^Department of Obstetrics, Gynecology and Reproductive Sciences, University of California, San Diego, La Jolla, CA, United States; ^2^Herbert Wertheim School of Public Health and Longevity Science, University of California, San Diego, La Jolla, CA, United States; ^3^Department of Medicine, University of California, San Diego, La Jolla, CA, United States; ^4^Department of Research and Health Promotion, San Ysidro Health Center, San Ysidro, CA, United States; ^5^Joint Doctoral Program in Public Health, San Diego State University/University of California, San Diego, San Diego, CA, United States; ^6^Department of Psychiatry, University of California, San Diego, La Jolla, CA, United States; ^7^Altman Clinical and Translational Research Institute Dissemination and Implementation Science Center, University of California San Diego, La Jolla, CA, United States; ^8^Child and Adolescent Services Research Center, San Diego, CA, United States

**Keywords:** vaccines, vaccine uptake, vaccine hesitancy, COVID-19, trusted sources of information, Latino, border community

## Abstract

**Background:**

COVID-19 vaccine uptake has been uneven, particularly across racial/ethnic and age groups. This study seeks to understand factors associated with COVID-19 vaccine uptake in a large cross-sectional sample of predominantly Latinos/Latinas individuals living near the US/Mexico border.

**Methods:**

Data are extracted from a 176-item survey conducted as part of a parent study focused on the co-creation of a COVID-19 testing program for underserved communities developed through a partnership between an academic institution and a Federally Qualified Health Center. The following participant variables were examined: health history, COVID-19 symptoms, COVID-19 testing and vaccine experiences, and perceptions of sources of health information. Participant characteristics were compared using chi-square tests. Multivariate logistic regressions were used for the final statistical model.

**Results:**

From 1 May 2021 to 30 April 2022, 4,964 adults, 66% of whom were identified as women, completed the survey. Approximately 80% of participants reported having received at least one COVID-19 vaccine. Female sex, older age, Hispanic/Latino(a) ethnicity, previous influenza vaccination, advanced education, and perceived elevated risk of COVID-19 were significantly (*p* < 0.05) associated with having received a COVID-19 vaccine. Regarding sources of health information, individuals who indicated they trust their doctor, healthcare provider, or the US government “a great deal” were more likely to have received a COVID-19 vaccine compared to individuals who indicated that they trusted these sources “not at all.” In contrast, those who reported having “a great deal” of trust in their faith leader or their social media contacts were significantly less likely to have received a COVID-19 vaccine than those who reported that they trusted these sources “not at all.”

**Conclusion:**

Sex, education, past influenza vaccination, perceived risk of COVID-19 infection, and trust in specific sources of information were correlated with the uptake of COVID-19 vaccination. Additional research is needed to better understand why this confluence of factors, particularly the unique findings about trusted sources of information, are associated with vaccine uptake. Understanding these associations, specifically within underserved, Latino/Hispanic communities, is an important first step to inform efforts aimed at increasing and sustaining COVID-19 vaccine uptake and adoption of other public health interventions.

## Background

### COVID-19 and vaccine uptake

In December 2020, Comirnaty (Pfizer-BioNTech) became the first COVID-19 vaccine available in the United States (US) and was initially approved for individuals aged 16 years or older under Emergency Use Authorization (EUA). This was followed by EUA approvals for Spikevax (Moderna) and the Janssen COVID-19 Vaccine (Johnson & Johnson) for those aged 18 years and older (1). Over time, these vaccines received approval for progressively younger age groups. Despite the availability of these COVID-19 vaccines, there have been variable rates of vaccination across different demographic groups (2, 3). Factors that influence vaccine uptake are multifactorial, including individual-level factors, but also structural, societal, and systemic (4, 5). Previous research has revealed that individual-level factors such as demographic characteristics and previous flu vaccination status reveal an association with COVID-19 vaccination rates (6, 7). Therefore, the following factors were evaluated to assess the differential factors associated with COVID-19 vaccine uptake: patient demographics, flu vaccination history, and perceptions of trusted sources of health information.

Disparities in COVID-19 vaccine uptake, particularly among underserved communities, have and may continue to exacerbate health inequities among populations who have been disproportionately affected by COVID-19 (8). Throughout the pandemic, the highest case rates of COVID-19 within San Diego County have been reported in the San Ysidro community (9). San Ysidro is a predominately low-income neighborhood located along the US/Mexico border where most of the residents identify as Hispanic/Latino(a) and are predominately monolingual Spanish speakers (10).

Understanding vaccine uptake patterns, trust in sources of health information, and reasons for vaccine hesitancy are key to building effective public health messaging (5, 11). The goal of this study was to identify individual-level factors associated with COVID-19 vaccine uptake in a large cross-sectional sample of San Ysidro residents.

## Methods

### Study design

Community-driven Optimization of COVID-19 testing to Reach and Engage underserved Areas for Testing Equity—in Women and Children (CO-CREATE) is a 2-year project funded by the National Institutes of Health Rapid Acceleration of Diagnostics for Underserved Populations (RADx-UP) program (12) with an overarching goal of eliminating the disparities experienced by underserved communities by directing efforts toward offering large-scale no-cost COVID-19 testing for children, pregnant women, and their families. UC San Diego partnered with a Federally Qualified Health Center (FQHC) in South San Diego and the Global Action Research Center, a social change organization, to co-create and demonstrate the impact of a COVID-19 testing program in the San Ysidro community, one of the most impacted areas in San Diego County. Recruitment for the CO-CREATE project began in May 2021 and is ongoing.

### Procedures

Individuals seeking a COVID-19 test at the partnering FQHC were invited to participate in the CO-CREATE study. The FQHC's patient population is predominantly Hispanic due to its proximity to the US–Mexico border. Testing was available most days of the week and was free of cost with no appointment needed. Staff members were all bilingual (English/Spanish). After the study participants consented to participate in the study, they were instructed to self-collect an anterior nares sample for COVID-19 testing and were invited to complete the study survey. Community members of all ages were eligible to participate with the exception of those who were unable to provide assent or consent (e.g., those with severe developmental delays or disabilities) or did not have a legal guardian who could consent on their behalf. Participants were permitted to return for follow-up study visits, which included repeat COVID-19 testing and a shorter survey. Surveys were available in either English or Spanish and could be completed using a paper form, an electronic tablet, or by accessing it online directly through a survey link from the Research Electronic Data Capture (REDCap) system (13). Responses for surveys that were completed on paper were entered into REDCap by project staff. To eliminate duplicate enrollments, identifying information was collected and verified by requiring participants to show proof of identification. Participants were compensated US$20 for their initial survey and US$10 for every return visit survey, both through gift cards.

### Ethics

This study received approval from the Institutional Review Board at the University of California, San Diego Human Research Protections Program, protocol number 210498. All participants provided informed consent acknowledging potential risks and the voluntary nature of study participation before participating in the study.

### Survey measures

The 176-item survey included Common Data Elements (CDEs) developed through Duke University as part of the RADx-UP program (14), and besides others, it included questions on socioeconomic status, comorbidities, influenza and COVID-19 vaccine status, trust in sources of information, and attitudes related to COVID-19 vaccine uptake and hesitancy. Information regarding trust in sources of information was gathered through a 4-point Likert scale with response options of “not at all,” “a little,” “somewhat,” and “a great deal” where “not at all” was the reference category in the analysis. None of the questions were required to be answered in the survey except for “date of birth” and “name.” In addition to self-reported vaccine status in the survey, study staff also asked participants about their COVID-19 vaccine status at the time of testing and recorded it in a separate file that was used to track study enrollment.

### Statistical analysis

For this analysis, we included only data of those participants aged > 18 years. The primary outcome of interest was reported by participants as a dichotomous yes/no variable. If participants initially reported being unvaccinated, but later reported at a return study visit that they had received a vaccine, they were classified as vaccinated for this analysis. Characteristics of participants who had received a COVID-19 vaccine vs. those who had not were compared using the chi-square tests for categorical variables. Variables attaining significance at α = 0.10 were considered candidates for inclusion in multivariate logistic regression models and were retained in the final multivariate model based on statistical significance and relationships among potential predictors (e.g., correlations, confounding, and interactions). Since the availability of COVID-19 vaccines changed over time, we included a linear term representing the month of participation in all multivariate models. All statistical analyses were conducted using STATA17 (StataCorp, College Station, TX).

## Results

Between 1 May 2021 and 30 April 2022, 6,955 individuals consented to and completed the CO-CREATE study survey. Of note, 29% (1,991) of all participants were < 18 years of age and were excluded from the analysis, resulting in 4,964 study participants.

A majority (57.5%) of the study participants included in this analysis reported speaking Spanish in their homes and 94.4% self-classified as Hispanic. The median age of participants included in this analysis was 44.0 years (interquartile range: 31.4, 58.0). Among the 4,964 adult participants, 246 (5.0%) did not report vaccine status, 3,951 (79.6%) reported having received at least one dose of the COVID-19 vaccine, and 767 (15.5%) reported not having received any COVID-19 vaccine doses. Table 1 shows our sample's demographics stratified by vaccination status.

**Table 1:** Distribution of participants' demographics based on COVID-19 vaccine status (*n* = 4,964).

	**Vaccine status**				
	**Unknown**	**Unvaccinated**	**Vaccinated**	**Total (** ***n*** **)**	***P*** **-value** ^*^
	246 (4.95%)	767 (15.5%)	3,951 (79.6%)	4,964 (%)	
**Participant demographics**, ***n*** **(%)**					
**Sex at birth**					**< 0.001**
Female	151 (61.4)	454 (59.2)	2,652 (67.1)	3,257 (65.6)	
Male	76 (30.9)	309 (40.3)	1,280 (32.4)	1,665 (33.5)	
Undefined^a^	19 (7.7)	4 (0.5)	19 (0.5)	42 (0.8)	
**Age category (years)**					**< 0.001**
18–24	13 (5.3)	132 (17.2)	440 (11.1)	585 (11.8)	
25–34	31 (12.6)	252 (32.9)	783 (19.8)	1,066 (21.5)	
35–44	58 (23.6)	152 (19.8)	685 (17.3)	895 (18.0)	
45–54	57 (23.2)	105 (13.7)	720 (18.2)	882 (17.8)	
55+	87 (35.4)	126 (16.4)	1,323 (33.5)	1,536 (30.9)	
**Language spoken at home**					**0.007**
Spanish	19 (7.7)	127 (16.6)	507 (12.8)	653 (13.2)	
English	85 (34.6)	415 (54.1)	2,356 (59.6)	2,856 (57.5)	
Other	1 (0.4)	12 (1.6)	61 (1.5)	74 (1.5)	
No response/missing	141 (57.3)	213 (27.8)	1,027 (26)	1,381 (27.8)	
**Ethnicity**					**0.005**
Not Hispanic/Latino(a)	54 (22.0)	51 (6.6)	171 (4.3)	276 (5.6)	
Hispanic/Latino(a)	192 (78.0)	716 (93.4)	3,780 (95.7)	4,688 (94.4)	
**Education level**					**< 0.001**
Less than high school	23 (9.3)	182 (23.7)	920 (23.3)	1,125 (22.7)	
High school, GED, or tech	22 (8.9)	290 (37.8)	1,381 (35)	1,693 (34.1)	
University degree or more	7 (2.8)	38 (5.0)	434 (11.0)	479 (9.6)	
Missing/no response	194 (78.9)	257 (33.5)	1,216 (30.8)	1,667 (33.6)	
**History of flu vaccination**					**< 0.001**
No	10 (2.7)	260 (33.9)	564 (14.3)	834 (16.8)	
Yes	25 (6.8)	249 (32.5)	2,255 (57.1)	2,529 (50.9)	
Missing/not reported	211 (57.2)	258 (33.6)	1,132 (28.7)	1,601 (32.3)	

* Tests of independence are performed after excluding unvaccinated and non-responses, and data included in Pearson's chi-square are denoted in bold.

a “Undefined” sex at birth includes those who declined to state, whose gender information is missing, or who identify as transgender, gender non-binary, gender queer, or intersex.

Individuals who indicated they had received a vaccine were then prompted to answer questions regarding their decision and experience receiving the COVID-19 vaccine. Over half (57%) of the respondents indicated that they decided to get vaccinated to keep their family safe. A similar proportion (59%) of vaccinated participants indicated that they experienced no difficulties obtaining a vaccine, and only 5% of individuals indicated that they had difficulty obtaining a vaccine appointment. Participants were also asked if there was anything that had made them worried about getting the vaccine. Nearly half (42%) of respondents indicated that there was nothing they were worried about when getting the vaccine, and nearly a quarter (24%) of vaccinated participants indicated that they were concerned about the side effects of the vaccine. Only few (8%) respondents indicated they did not know how well the vaccine works or were worried about the safety of the vaccine (6%) (see Table 2).

**Table 2:** Attitude(s) and belief(s) regarding the COVID-19 vaccine among respondents who are vaccinated (*n* = 3,951)^*^.

**Why did you decide to receive the COVID-19 vaccine?**	***n* (%)**
I wanted to keep my family safe	2,263 (57.3)
I wanted to keep myself safe	1,461 (37.0)
I wanted to keep my community safe	1,212 (30.7)
I wanted to feel safe around other people	1,035 (26.2)
I didn't want to get really sick from COVID-19	828 (21.0)
I believed life wouldn't go back to normal until most people got a COVID-19 vaccine	721 (18.3)
I have a chronic health problem, like asthma or diabetes	384 (9.7)
My doctor told me to get a COVID-19 vaccine	251 (6.4)
Other	133 (3.4)
**What made getting your COVID-19 vaccine difficult?**	***n*** **(%)**
There was nothing that made getting my vaccine difficult	2,334 (59.1)
It was difficult to get an appointment	217 (5.5)
Other	90 (2.3)
I didn't know where to go for my vaccination	70 (1.8)
I didn't know how to get an appointment for my vaccination	61 (1.5)
Vaccination locations were too far or hard to get to	48 (1.2)
I couldn't take time off work for my vaccination	44 (1.1)
I didn't have transportation to or from a vaccination location	30 (0.8)
I didn't have someone to watch my children/other people in my care while I went	26 (0.7)
I didn't have reliable technology to book my vaccination appointment	18 (0.5)
They didn't speak my language at the vaccination location	15 (0.4)
**Was there anything that made you worried when getting the vaccine?**	***n*** **(%)**
There was nothing I was worried about when getting my vaccine	1,668 (42.2)
I was concerned about side effects from the vaccine	930 (23.5)
I didn't know enough about how well a COVID-19 vaccine works	310 (7.9)
I didn't trust that the vaccine would be safe	241 (6.1)
I don't like needles	199 (5.0)
I was worried about catching COVID-19 by going to a vaccination location	97 (2.5)
I didn't believe the COVID-19 pandemic was as bad as some people say it is	63 (1.6)
I was worried about paying for it	41 (1.0)
I'm allergic to vaccines	32 (0.8)
It conflicts with my religious beliefs	23 (0.6)
I was worried about being asked to show documentation at a vaccine appointment	19 (0.5)

^*^Participants were instructed to “select all that apply.”

Among the 767 participants who indicated that they did not receive a COVID-19 vaccine, 31% reported that they were concerned about potential side effects of the vaccine, and 17% did not trust the vaccine to be safe. Approximately 15% of the participants also indicated “other” as a reason for not getting the vaccine; however, they did not share additional reasons for not having received a vaccine (see Table 3).

**Table 3:** Reported reason(s) for not receiving the COVID-19 vaccine among those who are not vaccinated (*n* = 767)^*^.

**What are some reasons you have NOT gotten the COVID-19 vaccine?**	***n* (%)**
I'm concerned about side effects from the vaccine	236 (30.8)
I don't trust that the vaccine will be safe	131 (17.0)
Other	111 (14.5)
I don't know enough about how well a COVID-19 vaccine works	63 (8.2)
I don't think vaccines work very well	48 (6.3)
I don't like needles	47 (6.1)
I'm not concerned about getting really sick from COVID-19	42 (5.5)
It conflicts with my religious beliefs	31 (4.0)
I don't believe the COVID-19 pandemic is as bad as some people say it is	23 (3.0)
I can't take time off work for a vaccination	20 (2.6)
I'm allergic to vaccines	20 (2.6)
I don't have transportation to or from a vaccination location	13 (1.7)
It is difficult to get an appointment	11 (1.4)
I don't know where to go for a vaccination	11 (1.4)
I don't know how to get an appointment for a vaccination	10 (1.3)
I don't want to pay for it	9 (1.2)
I don't have someone to watch my children/other people in my care while I go	7 (0.9)
I am worried about being infected with COVID-19 by going to a vaccination location	6 (0.8)
I don't have reliable technology to book a vaccination appointment	3 (0.4)
I am worried about being asked to show documentation at a vaccine appointment	3 (0.4)
They didn't speak my language at the vaccination location	1 (0.1)

^*^Participants were instructed to “select all that apply.”

**Figure 1:**
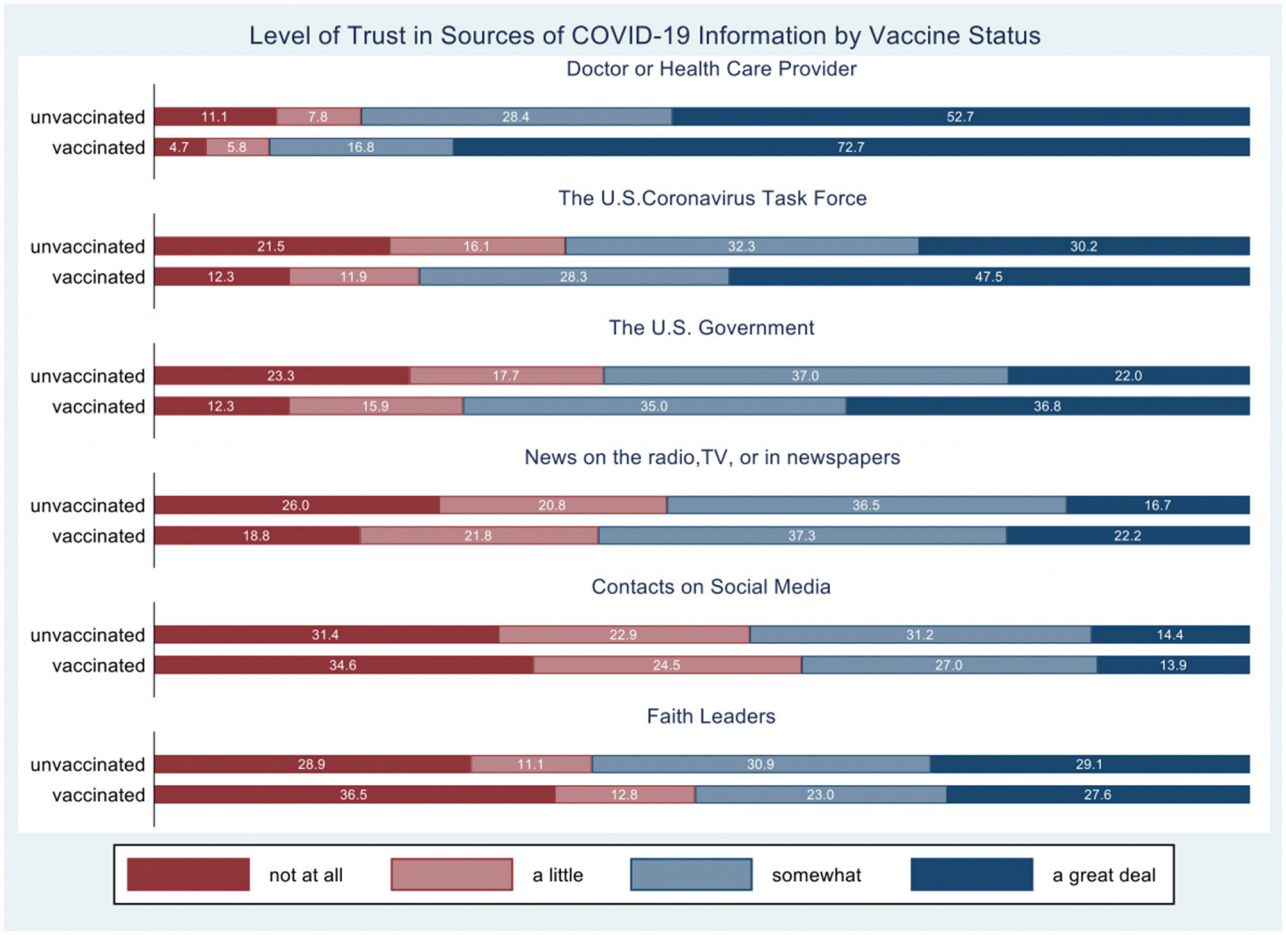
Degree of trust for COVID-19 information by source. The percentage of responses by category is presented separately for vaccinated and unvaccinated individuals.

Participants were also asked to rate how much they trusted different sources to provide correct information about COVID-19. Levels of trust were compared between vaccinated and unvaccinated individuals, for which the response rates were 53% and 63%, respectively. These responses are reported in Figure 1. A very small percentage of both vaccinated and unvaccinated individuals indicated they “not at all” trusted their healthcare provider; however, a markedly higher percentage of vaccinated participants indicate they trusted their healthcare provider “a great deal” (72.7%) than unvaccinated participants (52.7%). Similar, but less dramatic, results were observed for trust in the US Coronavirus Task Force, news, and the US government. In contrast, vaccinated participants were more likely to “not at all” trust faith leaders and contacts on social media compared to unvaccinated participants.

There were multiple demographic variables, health behaviors, and beliefs that were significantly associated with a participant's likelihood of receiving a COVID-19 vaccine. In the full model, see Table 4, the strongest correlate with a COVID-19 vaccine was a history of influenza vaccination (adjusted odds ratio [AOR] = 3.67, *p* < 0.01, 95% confidence interval [CI] = [2.97–4.55]).

**Table 4:** Logistic regression results examining factors associated with COVID-19 vaccine uptake.

**Variables**	**Categorical levels**	***P*-value**	**AOR**	**95% CI**
Sex at birth	Female [reference]			
	Male	0.00	0.67	(0.56, 0.80)
	Undefined/Missing	0.49	1.55	(0.45, 5.41)
Age category	18–24 [reference]			
	25–34	0.35	0.88	(0.68, 1.15)
	35–44	0.06	1.31	(0.99, 1.75)
	45–54	0.00	2.12	(1.56, 2.89)
	>55	0.00	2.84	(2.12, 3.81)
Ethnicity	Not Hispanic [reference]			
	Hispanic	0.02	1.55	(1.08, 2.23)
Education	Less than high school [reference]			
	High school/GED/tech school	0.11	1.21	(0.96, 1.54)
	University or graduate degree	0.00	2.48	(1.65, 3.72)
	Missing/no response	0.31	1.19	(0.85, 1.66)
History of flu vaccination	No [reference]			
	Yes	0.00	3.67	(2.97, 4.55)
	Missing/not reported	0.20	1.24	(0.90, 1.71)
Perceived risk of infection	Low risk [reference]			
	Medium risk	0.11	1.31	(0.94, 1.82)
	High risk	0.00	1.79	(1.24, 2.58)
	Don't know/no response	0.37	0.88	(0.67, 1.16)
Faith leader trust	Not at all [reference]			
	A little	0.44	0.84	(0.54, 1.30)
	Somewhat	0.02	0.64	(0.45, 0.93)
	A great deal	0.00	0.50	(0.35, 0.72)
	Missing/no response	0.01	0.60	(0.41, 0.88)
Social media contacts trust	Not at all [reference]			
	A little	0.21	0.80	(0.56, 1.13)
	Somewhat	0.12	0.75	(0.52, 1.08)
	A great deal	0.00	0.51	(0.32, 0.80)
	Missing/no response	0.79	0.94	(0.58, 1.51)
US government trust	Not at all [reference]			
	A little	0.02	1.67	(1.1, 2.53)
	Somewhat	0.00	2.00	(1.36, 2.96)
	A great deal	0.00	3.00	(1.95, 4.59)
	Missing/no response	0.03	1.75	(1.07, 2.86)
Healthcare provider trust	Not at all [reference]			
	A little	0.38	1.30	(0.73, 2.32)
	Somewhat	0.89	1.03	(0.64, 1.68)
	A great deal	0.00	1.98	(1.26, 3.09)
	Missing/no response	0.02	1.93	(1.11, 3.37)
Month of enrollment	Continuous	0.00	1.16	(1.11, 1.22)

AOR, adjusted odds ratio; CI, confidence interval.

Education and age were also positively and significantly associated with COVID-19 vaccination status. Participants with a college or graduate degree were 2.48 times more likely (AOR = 2.48, *p* < 0.01, 95% CI = [1.65–3.72]) to be vaccinated compared to those who did not have a high school degree. Participants aged 45–54 years were 2.12 times more likely (AOR = 2.12, *p* < 0.01, 95% CI = [1.56–2.89]) to be vaccinated against COVID-19 compared to those aged 18–24 years. Participants aged 55 years or older were 2.84 times more likely (AOR = 2.84, p < .01, 95% CI = [2.12–3.81]) to have been vaccinated compared to those aged 18–24 years. Sex was also correlated with COVID-19 vaccine status. Men were 33% less likely (AOR =.67, *p* < 0.01, 95% CI = [0.56–0.8]) to report having received any COVID-19 vaccination compared to women.

Trust in sources of information had varying associations with COVID-19 vaccination. Part of the survey asks participants to rate how much they trust certain sources of information on a 4-point Likert scale. “Not at all” is used as the reference point for all categories in the statistical analysis. There were eight sources of information that participants were prompted to rate their trust in. Trust in doctors or health providers had the strongest association with COVID-19 vaccine status; while there was a trend toward a positive association throughout all tested categories, statistical significance is reached only when comparing the “a great deal” category to the reference “not at all” category. Those who trusted doctors or healthcare providers “a great deal” were 1.98 times more likely (AOR = 1.98, *p* < 0.05, 95% CI = [1.26–3.09]) to be vaccinated against COVID-19 than those who responded with “not at all,” while controlling for the remaining variables. When asked about their trust in the US government, participants who responded with “a great deal” were 3 times more likely (AOR = 3.0, *p* < 0.01, 95% CI = [1.95–4.59]) to be vaccinated compared to those who responded with “not at all.” Participants who reported trust in their faith leader as “somewhat” were 36% less likely (AOR = 0.64, *p* < 0.05, 95% CI = [0.45–0.93]) to be vaccinated against COVID-19 compared to the reference cohort of those who answered with “not at all.” Furthermore, participants who reported having “a great deal” of trust toward their faith leader were 50% less likely (AOR = 0.5, *p* < 0.01, 95% CI = [0.35–0.72]) to be vaccinated against COVID-19 compared to the reference group that answered with “not at all.” Finally, participants who reported that they trust social media “a great deal” were 49% less likely (AOR = 0.51, *p* < 0.01, 95% CI = [0.32–0.8]) to report having received a dose of any COVID-19 vaccination compared to those who reported that they trusted social media “not at all.”

## Discussion

The adoption of public health interventions, such as the COVID-19 vaccine, is continuously challenged by the public's distrust and hesitancy of vaccines, often due to misinformation and personal health beliefs (16–18). These contributing factors are complex and multi-level, requiring multidisciplinary interventions to promote vaccine uptake. In this community-based study, multiple individual-level factors were significantly associated with COVID-19 vaccine uptake. Overall, most participants reported having received the COVID-19 vaccine (79.6%). Factors associated with the uptake of at least one COVID-19 vaccine included prior influenza vaccination, older age, higher education, Hispanic/Latino(a) ethnicity, and female sex. Furthermore, those who reported high trust in doctors and the US government also were more likely to have received at least one COVID-19 vaccine dose compared to those who did not trust these groups at all. The findings described in this study add to the growing but still insufficient literature on vaccine hesitancy research in under-represented minority populations, particularly the Hispanic/Latino(a) population. Furthermore, as a previous review article on minority COVID-19 vaccine uptake mentions, there is a need for more clinic-based studies among these populations to understand vaccine behavior better and to translate them into interventions (19).

Participants who reported having received an influenza vaccine in the past were 3.7 times more likely to have received a COVID-19 vaccine compared to those who did not report having ever received an influenza vaccine. A previous study mentions that people are more likely to be vaccinated against the influenza vaccine if they trust its effectiveness and safety (6). It is likely that those who believe in the effectiveness and safety of the influenza vaccine are also likely to trust other vaccines, such as the COVID-19 vaccine.

Men were significantly less likely to report having received any COVID-19 vaccination compared to women. This finding is consistent with other studies that compared the influenza vaccine uptake and sex (20). Given the predominantly Hispanic/Latino makeup of the participants, cultural influences may have impacted these gender differences. A study that examined Latino men and HPV vaccination reported that they are unlikely to be vaccinated against it, mentioning that one of the biggest reasons had to do with masculinity ideals (21). Higher education was also associated with a greater likelihood of COVID-19 vaccination. Our results are similar to Khubchandani and Macias' findings detailed in their 2021 review that those with less education were more likely to show vaccine hesitancy (19).

Self-reported trust in different sources of information was correlated with participants' likelihood of COVID-19 vaccination. First, there was a strong and significant positive correlation between trust in healthcare providers and the likelihood of being vaccinated against COVID-19. A study in rural Japan found that people tended to trust information that comes from medical facilities the most, which is comparable to our findings (6). Those who had less trust in their faith leader were more likely to have received a COVID-19 vaccine. In 2021, Chu et al. focused on the impact that Christian health providers have on encouraging unvaccinated Christians to get the COVID-19 vaccine and its success which suggests the importance of including faith leaders in community-led interventions due to their effectiveness in influencing religious people (22). Our findings highlight the importance of partnering with target communities for vaccine uptake campaigns, especially within religious communities; however, additional research-based strategies are needed to shape health marketing campaigns (23).

Future studies should focus on the dissemination and implementation of programs that will help bring appropriate vaccine information to marginalized communities, faith-based leaders, and monolingual speaking community members. These studies should focus on identifying subgroups within audiences that have a lower percentage of vaccine uptake.

We offer several recommendations for public health researchers and practitioners based on our study findings. First, we recommend consideration of a public health and/or dissemination and implementation science model, theory, or framework to guide the design and evaluation of COVID-19 prevention and treatment programs. As described in our study, identification of populations at the highest risk for non-compliance with preventative measures, such as vaccination, is key to developing culturally and linguistically targeted health education programs. In particular, within religious communities, identification of and partnership with community leaders to ensure accurate vaccine information diffusion among community members may reduce the risk of vaccine misinformation and vaccine hesitancy (24, 25). Use of theories such as the Theory of Reasoned Action (26) or the Health Belief Model (27) may help understand how social and environmental factors can contribute to the community's health perception and behavior of vaccine uptake (28) and enable future studies to develop more effective and sustainable health interventions for vaccine uptake. A relevant dissemination and implementation science model to consider is the Diffusion of Innovation Theory (15), a method of disseminating vaccine information and vaccines among communities with high vaccine hesitancy.

### Strengths and limitations

The primary limitation of our study was that the data used for this analysis were collected solely from self-selected participants who consented to participate in the study and were not a representative sample of all participants who were tested. Although vaccine status was noted for every individual who tested, only those who consented to the study were included in this study. Individuals who opted to test and to participate in public health research may differ from those who did not opt for testing and research participation, thus creating a potential sample selection bias. In addition, survey fatigue may have impacted the data collected as we observed that the questions positioned toward the end of the survey had a lower response rate. We recommend randomization of the order of questions to distribute the likelihood of missing data more evenly for studies with longer survey instruments. Finally, we acknowledge the potential of reporting bias as we did not require proof of vaccination status from participants but instead collected only self-reported data.

Balanced with these study limitations are several strengths. First, the survey was offered in both English and Spanish with the option of a paper survey for participants who were not comfortable using an electronic device. With a large monolingual Spanish-speaking community, providing materials in both English and Spanish was essential to gaining participant trust and participation. Second, a verbal survey was an option for participants who were unable to read or write comfortably enough to complete the survey. Finally, the CO-CREATE study opened an internship through UC San Diego to provide bilingual students and those from the target community opportunities to actively participate in participant consent and enrollment, data collection and analysis, and dissemination of study findings.

## Conclusion

Vaccine hesitancy is complex and is influenced by a variety of factors. In our study population, previous influenza vaccination, older age, female sex, higher education, and higher levels of trust in healthcare providers and government organizations were associated with increased uptake of COVID-19 vaccines. This underscores the importance of considering multiple individual-level factors when creating, implementing, and evaluating COVID-19 public health interventions, especially those in underserved, Spanish-speaking communities. Data collected through the CO-CREATE project along with future research on vaccine intentions and behavior will help to create future interventions aimed at addressing an individual's hesitancy in vaccinations and potentially also improve the overall trust in science and research.

## Data availability statement

The datasets presented in this article are not readily available because the project is still in progress. Requests to access the datasets should be directed to LL, llaurent@health.ucsd.edu.

## Ethics statement

The studies involving human participants were reviewed and approved by UC San Diego Institutional Review Board. The patients/participants provided their written informed consent to participate in this study.

## Author contributions

AL, AE, BR, MB, ST-C, AC, and IV contributed to data collection and data cleaning. LL, LS, NS, and BR contributed by designing the study and editing multiple drafts of the paper. MS contributed by leading the data and results portion of the paper. All authors revised the paper multiple times and approved the final version.
